# A Direct Comparison of Physical Versus Dihydrocapsaicin-Induced Hypothermia in a Rat Model of Traumatic Spinal Cord Injury

**DOI:** 10.1089/ther.2021.0013

**Published:** 2022-06-07

**Authors:** Amrita Sarkar, Kevin T. Kim, Orest Tsymbalyuk, Kaspar Keledjian, Bradley E. Wilhelmy, Nageen A. Sherani, Xiaofeng Jia, Volodymyr Gerzanich, J. Marc Simard

**Affiliations:** ^1^ Department of Neurosurgery, University of Maryland School of Medicine, Baltimore, Maryland, USA.; ^2^ Department of Neurosurgery, Pathology and Physiology, University of Maryland School of Medicine, Baltimore, Maryland, USA.

**Keywords:** SCI, hypothermia, dihydrocapsaicin

## Abstract

Spinal cord injury (SCI) is a devastating neurological condition with no effective treatment. Hypothermia induced by physical means (cold fluid) is established as an effective therapy in animal models of SCI, but its clinical translation to humans is hampered by several constraints. Hypothermia induced pharmacologically may be noninferior or superior to physically induced hypothermia for rapid, convenient systemic temperature reduction, but it has not been investigated previously in animal models of SCI. We used a rat model of SCI to compare outcomes in three groups: (1) normothermic controls; (2) hypothermia induced by conventional physical means; (3) hypothermia induced by intravenous (IV) dihydrocapsaicin (DHC). Male rats underwent unilateral lower cervical SCI and were treated after a 4-hour delay with physical cooling or IV DHC (∼0.60 mg/kg total) cooling (both 33.0 ± 1.0°C) lasting 4 hours; controls were kept normothermic. Telemetry was used to monitor temperature and heart rate during and after treatments. In two separate experiments, one ending at 48 hours, the other at 6 weeks, “blinded” investigators evaluated rats in the three groups for neurological function followed by histopathological evaluation of spinal cord tissues. DHC reliably induced systemic cooling to 32–33°C. At both the time points examined, the two modes of hypothermia yielded similar improvements in neurological function and lesion size compared with normothermic controls. Our results indicate that DHC-induced hypothermia may be comparable with physical hypothermia in efficacy, but more clinically feasible to administer than physical hypothermia.

## Introduction

Each year, traumatic spinal cord injury (SCI) afflicts 10–83 patients per million worldwide (Wyndaele and Wyndaele, 2006). In the United States, the annual incidence of SCI is ∼40 patients per million (Devivo, 2012). Acute SCI can leave patients with profound long-term neurological symptoms and other deficits, including paralysis, bowel and bladder dysfunction, spasticity, autonomic dysregulation of cardiovascular and respiratory function, osteoporosis, and chronic pain (Sezer *et al.*, 2015). Effective treatments that can be applied during the immediate postinjury phase remain elusive.

Hypothermia has been extensively studied in animal models of traumatic SCI (Wang and Pearse, 2015; Martirosyan *et al.*, 2017), with the first reports appearing over half a century ago (Albin *et al.*, 1967, 1968). Two systematic reviews and meta-analyses have documented a clear benefit of hypothermia in animal models of SCI (Batchelor *et al.*, 2013; Yousefifard *et al.*, 2020). The more recent review examined 30 separate experiments from 20 reports on rats and found that hypothermia has a significantly positive effect on locomotion (Yousefifard *et al.*, 2020). Subgroup analyses showed that systemic hypothermia, more so than local hypothermia, significantly improves recovery of locomotion, and that hypothermia is more likely to affect motor recovery if its duration was 2–8 hours and the target temperature was 32–35°C. Notably, most preclinical studies have examined the effects of hypothermia when treatment was begun shortly after or within 1 hour of the trauma (Yousefifard *et al.*, 2020). However, systemic hypothermia instituted at the more clinically relevant time of 4 hours after trauma also has been shown to have significant efficacy (Wells and Hansebout, 1978; Hosier *et al.*, 2015).

In humans, several early-stage clinical studies have supported the use of systemic hypothermia (a.k.a. targeted temperature management) as a safe and beneficial neuroprotective intervention in SCI (Ahmad *et al.*, 2014; Martirosyan *et al.*, 2017). Currently, a large randomized clinical trial is underway, “Systemic Hypothermia in Acute Cervical Spinal Cord Injury” (NCT02991690). The predominant method of cooling used in humans is endovascular cooling via an intravenous (IV) catheter (Polderman and Callaghan, 2006), which may be cumbersome to implement, cannot be used outside a hospital setting, and carries a risk of thromboembolic complications (Andremont *et al.*, 2018; Wang *et al.*, 2018). Hypothermia induced by an IV drug infusion is an alternative method that may obviate some of the constraints and potential complications associated with physical devices.

Capsaicin is the purified compound responsible for the pungent activity of red peppers (*Capsicum annuum*) and has been shown to produce a deep fall in body temperature when administered to rats (Miller *et al.*, 1982; Hayes *et al.*, 1984) and other animals (Szolcsányi, 2014). Dihydrocapsaicin (DHC), the capsaicinoid most commonly studied (Feketa and Marrelli, 2015), has been shown to reliably induce mild-to-moderate hypothermia following IV administration in mammals via its action as an agonist at transient receptor potential cation channel subfamily V member 1 (TRPV1) receptors (Fosgerau *et al.*, 2010b; Cao *et al.*, 2014). DHC has been used to induce pharmacological hypothermia in animal models of cardiac arrest (Junyun *et al.*, 2016; Zhong *et al.*, 2018) and cerebral ischemia (Cao *et al.*, 2014, 2017; Janyou *et al.*, 2017) with favorable results, but has not been examined in models of SCI.

Here, we used a rat model of cervical SCI to compare the effects of hypothermia (33.0 ± 1.0°C) induced by IV DHC versus hypothermia induced by conventional physical means (cold fluid). Hypothermia treatments were begun at the clinically relevant time of 4 hours, as in our previous report (Hosier *et al.*, 2015).

## Methods

### Ethics statement

All methods and data are reported with consideration for the guidelines provided by *Animals in Research: Reporting in Vivo Experiments (ARRIVE) and Minimum Information About a Spinal Cord Injury Experiment (MIASCI)* (Kilkenny *et al.*, 2010; Lemmon *et al.*, 2014). We certify that all applicable institutional and governmental regulations concerning the ethical use of animals were followed during the course of this research. Animal experiments were performed under a protocol approved by the Institutional Animal Care and Use Committee of the University of Maryland, Baltimore, and in accordance with the relevant guidelines and regulations as stipulated in the U.S. National Institutes of Health Guide for the Care and Use of Laboratory Animals. All efforts were made to minimize the number of animals used and their suffering.

### Subjects

Male Long-Evans rats (10–12 weeks; 275–325 g; Envigo, Frederick, MD) were maintained on a 12-hour light/dark cycle and had free access to water and standard rat chow.

### Time line of experiments

Rats underwent intra-abdominal implantation of a telemetry probe (details below) followed the next day by SCI surgery (details below). Rats were assigned to one of three groups: (1) DHC cooling after SCI; (2) physical cooling after SCI; (3) normothermia after SCI, with all treatments delayed 4 hours, and lasting 4 hours (treatment details below).

Experiments were performed in several successive series. Long-term experiments (outcomes at 6 weeks) were divided into two series, with two rats processed each day. In *series 1*, 16 rats (eight per group) underwent SCI and 4 hours later were randomly assigned to undergo either physical or DHC hypothermia lasting 4 hours, followed by 4 hours of rewarming. Random treatment assignments were based on a random number generator. In *series 2*, eight rats underwent SCI and 4 hours later, all were actively maintained normothermic for 4 hours. Short-term experiments (outcomes at 48 hours) were conducted as a single series, *series 3*, with two to three rats processed each day. In *series 3*, 21 rats (seven per group) underwent SCI and 4 hours later were randomly assigned to maintain normothermia or undergo either physical or DHC hypothermia lasting 4 hours, followed by 4 hours of rewarming. In all series, during the 4 hours between SCI surgery and start of treatment, animals were gently warmed using a warm water heating pad or infrared heating lamps to minimize spontaneous hypothermia.

After completion of individual series, additional rats were processed to replace any that died prematurely, to maintain a balanced design for short-term (seven rats/group) and long-term (eight rats/group) experiments. Early mortality (<24 hours postoperatively), which we attributed to prolonged, repeated anesthesia (on day −1, for telemetry probe implantation; on day 0, first for SCI surgery followed by reanesthesia at 4 hours for the 4-hour treatment plus 4-hour rewarming; at day 1, for removal of the implanted telemetry probe), was highest in the normothermia group (*n* = 9), and was similar among the hypothermia groups (*n* = 3 and 4 in *series 1* and *series 2*, respectively); late mortality (>24 hours) was 0 or 1 per group.

For long-term outcomes, rats in *series 1* and *series 2* were evaluated during the first week for ptosis and urinary function, after which they were evaluated weekly for 6 weeks for modified Basso, Beattie, and Bresnahan (mBBB) scores, performance on the accelerating rotarod, beam balance, grip strength, and body mass (details below). After completing the functional evaluations at week 6, rats were euthanized, and the spinal cords were processed to quantify lesion volumes and spared tissues.

For short-term outcomes, rats in *series 3* were evaluated at 48 hours for mBBB scores, then were euthanized, and the spinal cords were processed for histopathology.

Different investigators were responsible for different functions, including on day 0, surgery for SCI and IV catheter placement (O.T.), and administering hypothermia treatments (A.S., K.T.K., O.T., B.E.W., and N.A.S.), and on day 1 and later, neurological and urological evaluation (A.S., K.T.K., K.K., B.E.W., and N.A.S.), and measurement of lesion volume (A.S.) and histopathology (B.E.W. and N.A.S.). On day 0 (surgeries and treatments), group identity could not be concealed. On day 1, rats were transferred to cages with masked identity by a scientist (V.G.) not involved in outcome evaluations. From day 1 to the end of the study, evaluations were performed by investigators who were “blinded” to treatment group identity.

### Sample size calculation

We based the sample size calculation on a previous study that used the same model of SCI to examine the effect of hypothermia (Hosier *et al.*, 2015). In that study, mean lesion volumes were 4.8 versus 3.1 mm^3^ (standard deviation_pooled_, 1.0 mm^3^; *n* = 8) for control versus hypothermia, respectively, which yields an effect size (Cohen's *d*) of 1.7. Sample size calculation for a two-sample comparison (*α* = 0.05; two tailed), an effect size of 1.7, and a power of 80% indicated a minimum sample size of 7 per group, similar to other studies with this model (Simard *et al.*, 2012b).

### Telemetry probe implantation

Implantable radiotelemetric probes (G2 HR E-Mitter^®^ series; Starr Life Sciences; model ER-4000) and VitalView telemetric software were used to monitor core body temperature and heart rate before, during, and after treatment. General anesthesia was induced and maintained using isoflurane, and surgery was performed using aseptic techniques to implant an E-Mitter in the peritoneal cavity. The probe body was loosely sutured to the abdominal wall to prevent migration. The negative lead was pushed through a small incision in the external oblique muscle. The head of the negative lead was threaded subcutaneously and sutured loosely to the right pectoralis superficialis muscle through a small incision near the clavicle. The positive lead was pushed through a second small incision in the external oblique muscle, threaded subcutaneously, and sutured loosely to a chest muscle on the left side of the chest near the diaphragm. For monitoring, individual rats were placed in plastic cages on telemetric receivers (ER-4000; Starr Life Sciences), and VitalView software (Starr Life Sciences) was used to monitor core body temperature and heart rate every 12 seconds. Implanted telemetry devices were removed on day 1 after SCI.

### Rat model of lower cervical hemicord contusion

A unilateral impact to the cervical spinal cord at C7 was calibrated to produce ipsilateral but not contralateral primary hemorrhage (Simard *et al.*, 2010, 2012a). Rats were anesthetized (ketamine, 60 mg/kg plus xylazine, 7.5 mg/kg, IP) and the head was mounted in a stereotaxic apparatus (Stoelting, Wood Dale, IL). Surgery was performed using aseptic techniques and magnification (Operating Microscope with Co-Axial Lighting; Zeiss, NY). The dorsal surface of the spine was exposed from C3 to T3 via a 4 cm midline incision and subperiosteal dissection of the paraspinous muscles. At C7, the spinous process, part of the right lamina, the entire left lamina, and the dorsal half of the left pedicle were removed using a 1.9-mm diamond burr (no. HP801-019; DiamondBurs.Net) and a high-speed drill, with care taken to avoid mechanical and thermal injury to the underlying dura and spinal cord. The dura was exposed by sharply removing the interlaminar ligaments rostral and caudal to the C7 lamina and any remaining portions of ligamentum flavum. A guide tube containing the impactor (1.55 mm tip diameter, 57 mm length, 1.01 g) was angled 5° medially, and the impactor was positioned on the dura near the left C8 nerve root using the stereotaxic manipulator arm. As previously (Simard *et al.*, 2010, 2012a), the caudal edge of the impactor was placed in line with the caudal edge of the C8 nerve root at the axilla, and the medial edge of the impactor was placed 0.3 mm medial to the dorsolateral sulcus. After impactor positioning, a 10 g weight was released inside the guide tube from a height of 25 mm and removed immediately after the impact. The impact caused an immediate, forceful flexion of the trunk and 70–90° flexion of the ipsilateral knee, which had been extended before impact, and resulted in a hemorrhagic contusion evident on the surface of the cord involving the ipsilateral but not the contralateral sulcus, as observed under the microscope through the translucent dura. Sterile normal saline (NS; 1 mL) was used to flush the surgical site. Paraspinous muscles were repositioned and the skin was sutured. After SCI surgery, rats were administered 10 mL sterile, glucose-free NS subcutaneously, were recovered from anesthesia, and were gently warmed using a warm water heating pad or infrared heating lamps to maintain approximate normothermia.

### Physical hypothermia

At 4 hours after SCI surgery, rats were reanesthetized with isoflurane, and moderate hypothermia (33 ± 1°C core temperature) was induced for 4 hours as described (Lo *et al.*, 2009; Hosier *et al.*, 2015). Rats were transferred to the cooling system that comprised a water blanket and pump (HTP WD-020 and HTP-1500; Adroit Medical Systems Corp., Loudon, TN), with the pump reservoir filled with ice water. The rats were covered with a thermal blanket to stabilize their temperature. After 4 hours of cooling, the water blanket temperature was gradually raised to warm the animals at 1°C/hour until normothermic (36–37°C) (Lo *et al.*, 2009).

### DHC hypothermia

At 4 hours after SCI surgery, rats were reanesthetized with isoflurane, and moderate hypothermia (33 ± 1°C core temperature) was induced pharmacologically for 4 hours. Under sterile conditions, a 0.94 mm catheter (R-JVC-M37; Braintree Scientific, Inc., MA) was inserted into the right jugular vein via a small neck incision and secured with a suture. This catheter was connected to a 5 mL syringe and loaded with a sterile solution of 2% dimethyl sulfoxide (DMSO) in NS containing 0.225 mg/mL DHC (Cayman Chemical, MI). A syringe pump (NE-300, New Era Pump Systems, NY) was used to infuse this solution IV at 1.0 mL/hour for 15 minutes, then reduced to ∼0.15 mL/hour to obtain and maintain temperature in the target zone for 4 hours. For a 300 gm rat, this infusion schedule resulted in the administration of 0.60 mg/kg of DHC total, and 59 mg/kg of DMSO total, over the course of 4 hours. Catheter incision sites were covered with sterile gauze soaked in sterile NS during cooling. After cooling, the jugular catheter was removed and the incision was sutured. Animals were rewarmed gradually using a heated water blanket (HTP-1500; Adroit Medical Systems) at 1°C/hour until normothermic (36–37°C).

### Normothermic controls

At 4 hours after SCI surgery, rats were reanesthetized with isoflurane, and normothermia (36–37°C core temperature) was induced and maintained using a pump and water blanket system (HTP-1500; Adroit Medical Systems) containing warm water.

For animals in all groups, anesthesia and the target temperature were maintained for 4 hours while monitoring core temperature and heart rate telemetrically. After treatments were complete, animals were administered another 10 mL sterile, glucose-free NS subcutaneously, following which they were recovered from anesthesia. Telemetric monitoring was continued throughout treatment, rewarming, and overnight. Subsequently, animals were housed singly until euthanasia. After treatments, rats were supplied with easily accessible water and a purified high-calorie supplement (DietGel Boost; Clear H_2_O, Portland, ME).

### Ptosis

The presence of ptosis involving the ipsilateral and contralateral eye was assessed on days 1, 4, and 7. A score of 1 was used to denote the presence of ptosis, and 0 for no ptosis.

### Urinary function

On two to three occasions per day, the bladder was palpated to determine fullness. If full, manual compression was applied to initiate emptying (Credé's maneuver). The appearance of the urine, whether grossly bloody (hemorrhagic cystitis) or not, was recorded.

### mBBB scores

BBB scores were determined as described (Basso *et al.*, 1995), except that modifications were introduced to allow a more accurate assessment of the functional asymmetry associated with hemicord injury, as we previously described (Simard *et al.*, 2012a). The original report (Basso *et al.*, 1995) provides for scoring of the right and left hind limbs separately, with the final score for the animal being based on the two scores combined. Here, because we were inducing hemicord injuries, we maintained the two scores separately, which we refer to as mBBB scores (Simard *et al.*, 2012a). The method for scoring up to and including a score of 11 is identical for mBBB and BBB. For mBBB scoring of coordination, forelimb/hind limb coordination was operationally defined as a one-to-one correspondence between the forelimb and ipsilateral hind limb steps on each side. For scores above 19, both the right and left sides were credited for “tail consistently up” and for “trunk stability.” mBBB assessments were video recorded on days 4 and 7 following SCI and weekly thereafter for 6 weeks, and scored by two independent investigators, with the lowest score used for data analysis.

### Accelerating rotarod

Performance on the accelerating rotarod (IITC Life Science, Woodland Hills, CA) was measured as described (Hamm *et al.*, 1994). Initially the drum was rotated at 5 revolutions per minute (rpm), reaching a maximum of 45 rpm over 100 seconds. Each rat underwent three trials with 20 minutes of rest between trials. The average time to fall off the drum was reported for each animal. Animals received a score of 0 if they were unable to make any visible effort to remain on the drum.

### Beam balance

Performance on the beam balance was evaluated as described (von Euler *et al.*, 1996), with scores determined as follows: 0—falls off; 1—hangs on; 2—stands on beam but 1 or 2 legs slip off; 3—stands on beam; and 4—walks on beam.

### Grip strength

A rodent grip strength meter (Harvard Apparatus, MA) was utilized to measure grip strength in the ipsilateral, contralateral, and both the hind paws together. Each animal was held under the forelimbs and allowed to grip the meter briefly, then pulled away to generate a force measurement. Measurements were taken in triplicate and averaged.

### Body mass

Body mass was assessed on days 0, 2, 4, and 7 after trauma and weekly thereafter for 6 weeks.

### Histopathology

Rats were deeply anesthetized and underwent transcardial perfusion with NS (60 mL), followed by 10% neutral buffered formalin (50 mL; Sigma-Aldrich). Spinal cords were harvested from the skull base to midthorax. Treatment identities remained blinded throughout lesion analysis.

The spinal cords of rats from the short-term experiment were processed for fluorescence immunohistochemistry and terminal deoxynucleotidyl transferase dUTP (TUNEL) as we described (Gerzanich *et al.*, 2019). Spinal cords were immersion fixed in formalin for an additional 24 hours and cryopreserved in 30% sucrose. Cryosections (12 μm) collected at the epicenter of the injury were processed for TUNEL according to kit instructions (C10617; Thermo Fisher Scientific, Inc.), followed by immunolabeling with CY3-conjugated monoclonal anti-glial fibrillary acidic protein (GFAP) antibody (1:500; C9205; Sigma-Aldrich). Lesion boundaries were identified as the areas that were TUNEL+/GFAP–, and planimetric contours were drawn in Photoshop CS6 to quantify lesion areas at the epicenter.

The spinal cords of rats from the long-term experiment were paraffin embedded and processed to determine lesion size and spared tissue. Serial sections (10 μm) were prepared from 1.5 cm spinal cord segments centered around the injury epicenter. Sections were collected every 250 μm for all spinal cord samples and were mounted on glass slides for staining with hematoxylin and eosin (H&E). Following coverslipping and scanning at 2400 dpi using a flatbed scanner, we used Photoshop CS6 (v 13.0 × 64; Adobe Systems, Inc., San Jose, CA) to view each image enlarged approximately 40 × . Lesion boundaries were identified by distinct reductions in H&E staining in injured areas, and planimetric contours were drawn in Photoshop CS6 around lesions to quantify lesion areas and spared tissue in each section (Jakeman, 2012). Lesion and spared tissue volumes (in mm^3^) were calculated as the sum of areas per spinal cord section multiplied by 0.25 mm.

### Data analysis

Data are presented as mean ± standard error. Data were analyzed using Fisher's exact test, *χ*^2^, analysis of variance (ANOVA), or repeated-measures ANOVA, as appropriate, with *post hoc* Bonferroni correction. Statistical tests were performed using Origin Pro (V8; OriginLab, North Hampton, MA). Significance was assumed if *p* < 0.05.

## Results

### Temperature and heart rate

**FIG. 1.**
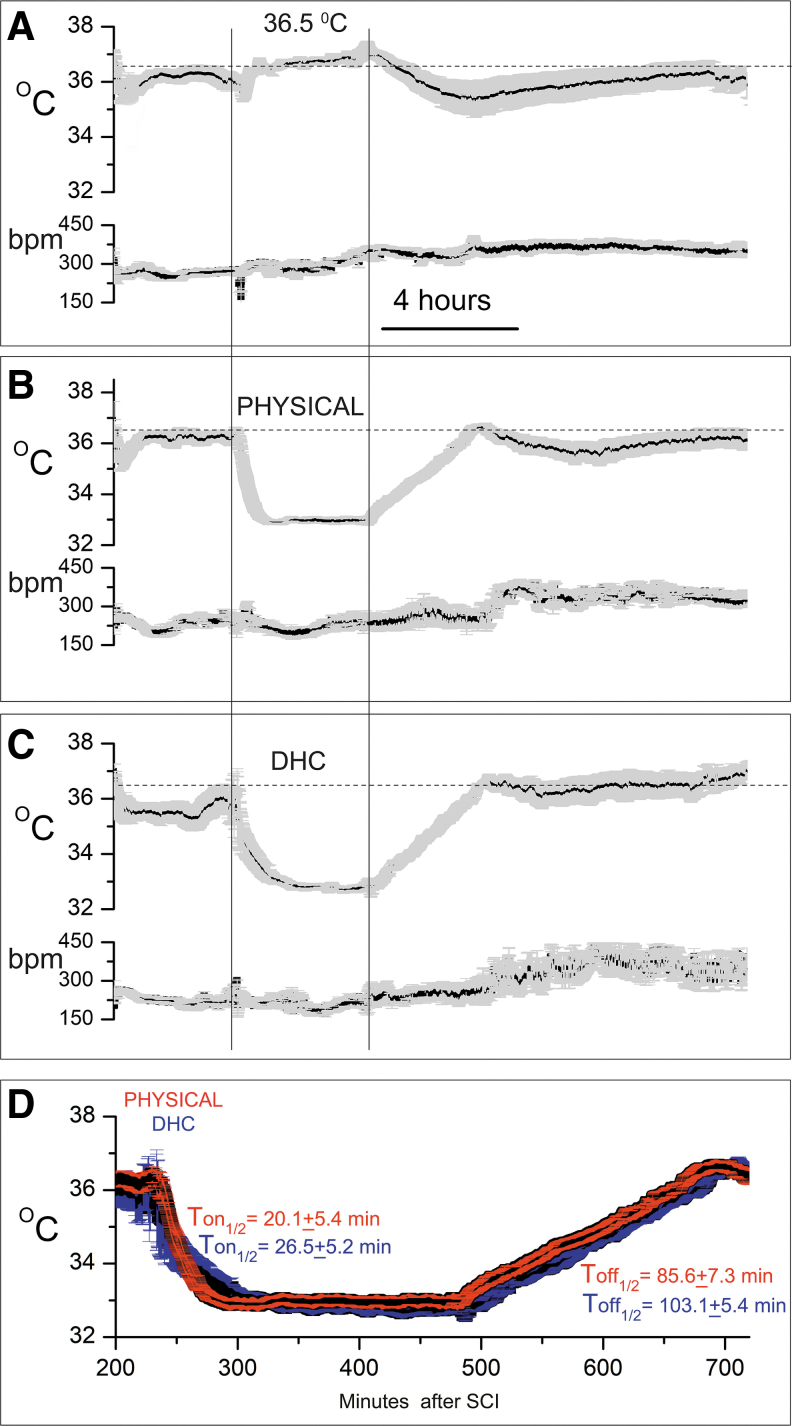
Temperature and heart rate after SCI. **(A–C)** Telemetric recordings (mean ± SE) of temperature and heart rate following lower cervical hemicord contusion in rats treated at 4 hours with normothermia **(A)**, with PH **(B)**, or with DHC-induced hypothermia **(C)**, before and during treatments, during gradual rewarming (each period lasting 4 hours), and during subsequent hours overnight; bpm, beats per minute; 15 rats per group. **(D)** Superimposed temperature records during physically induced (*red*) and DHC-induced (*blue*) hypothermia, showing similar temperature profiles for both treatments. DHC, dihydrocapsaicin; PH, physically induced hypothermia; SCI, spinal cord injury; SE, standard error.

The heart rate was stable throughout in all groups (Fig. 1).

At 4 hours after SCI surgery, rats in the control group were treated to maintain normothermia using a pump and water blanket system containing warm water (Fig. 1A). After 4 hours, active warming ceased, and core temperature dropped spontaneously before gradually recovering.

At 4 hours after SCI surgery, rats assigned to physical hypothermia underwent cooling to 33 ± 1°C for 4 hours using a pump and water blanket system containing ice water. After 4 hours of cooling, the water blanket temperature was gradually raised to warm the animals at 1°C/hour until normothermic (36–37°C) (Fig. 1B). Subsequent spontaneous cooling was transient and mild.

At 4 hours after SCI surgery, rats assigned to pharmacological hypothermia underwent cooling to 33 ± 1°C for 4 hours using an IV infusion of DHC. After 4 hours of cooling, the DHC infusion was stopped and animals were rewarmed gradually on a warm water blanket at 1°C/hour until normothermic (36–37°C) (Fig. 1C). Subsequent spontaneous cooling was minimal.

As implemented, the two hypothermia treatments resulted in similar temperature profiles during treatment (Fig. 1D). The times to reach half the target temperature (T-on_1/2_), 20.1 ± 5.4 and 26.5 ± 5.2 minutes for physical and DHC-hypothermia, respectively, were not statistically different. Similarly, the times to reach half the rewarming temperature (T-off_1/2_), 85.6 ± 7.3 and 103.1 ± 5.4 minutes for physical and DHC-hypothermia, respectively, were not statistically different. Mean temperatures at the 2-hour midpoint of the target temperature, 32.9. ± 0.1 and 32.8 ± 0.1°C for physical and DHC-hypothermia, respectively, were not statistically different.

### Experiment with short-term outcomes

#### mBBB scores

The model we studied with unilateral cervical SCI results in potentially asymmetric hind limb locomotor function, which we assessed using an mBBB scoring system adjusted to maintain the score for each hind limb separately (see Methods and Simard *et al.*, 2012a).

**FIG. 2.**
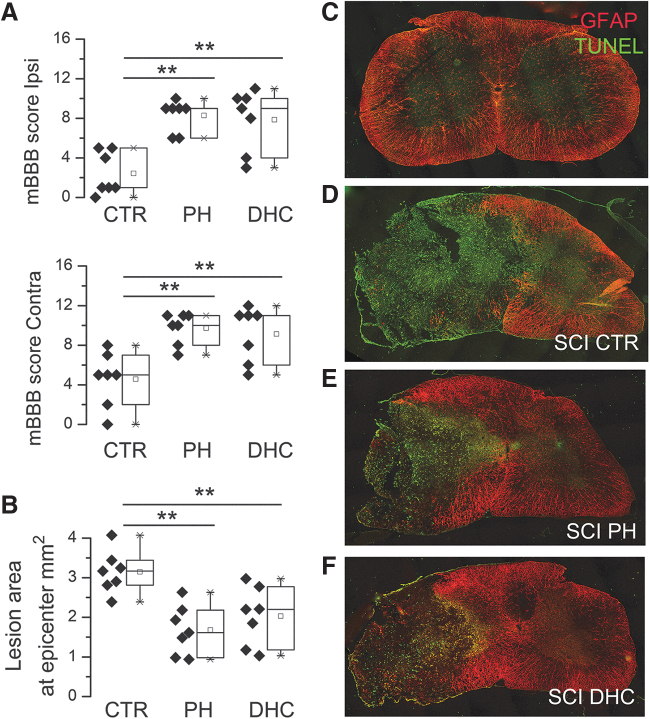
Early outcomes at 48 hours after traumatic SCI. **(A)** Ipsilateral and contralateral mBBB scores in rats treated at 4 hours with normothermia (CTR), with PH, or with DHC-induced hypothermia (DHC) at 48 hours after trauma; for both panels, values for the two hypothermia groups were significantly different from normothermic controls but not different from each other (*p* < 0.001). **(B–F)** Representative images **(C–F)** and areas **(B)** of lesions at 48 hours for the three experimental groups **(D–F)** plus an uninjured naive animal **(C)**; sections labeled for TUNEL (*green*) and GFAP (*red*); seven rats per group; **p < 0.01. CTR, control; DHC, dihydrocapsaicin; GFAP, glial fibrillary acidic protein; TUNEL, terminal deoxynucleotidyl transferase dUTP; mBBB, modified Basso, Beattie, and Bresnahan; PH, physically induced hypothermia; SCI, spinal cord injury.

Testing at 48 hours after trauma revealed that mBBB scores for the ipsilateral and contralateral hind limbs were similar for both the hypothermia modalities, and that both were significantly better than scores for the normothermic controls (Fig. 2A).

#### Histopathology

Lesion areas at the epicenter, assessed at 48 hours after the trauma, were defined as the areas that were TUNEL+/GFAP– (Fig. 2C–F). In the normothermic control group, the lesion area at the epicenter measured 3.4 ± 0.14 mm^2^ was the largest among the three groups (Fig. 2B, D), and was slightly larger than the area reported previously in the same model at 6 weeks (Hosier *et al.*, 2015). In the two hypothermia groups, lesion areas were significantly less, 1.7 ± 0.23 mm^2^ and 2.0 ± 0.28 mm^2^ (Fig. 2B, E, F), were not significantly different from each other, and were slightly larger than the area measured at 6 weeks in the same model treated with physical hypothermia (Hosier *et al.*, 2015).

### Experiment with long-term outcomes

#### Ptosis

Injury to the lower cervical spinal cord can extend into the upper thoracic cord, which houses preganglionic neurons of the sympathetic nervous system that control eye opening. The development of ipsilateral ptosis signals downward expansion of the lesion; the development of bilateral ptosis signals downward and contralateral expansion of the lesion.

**FIG. 3.**
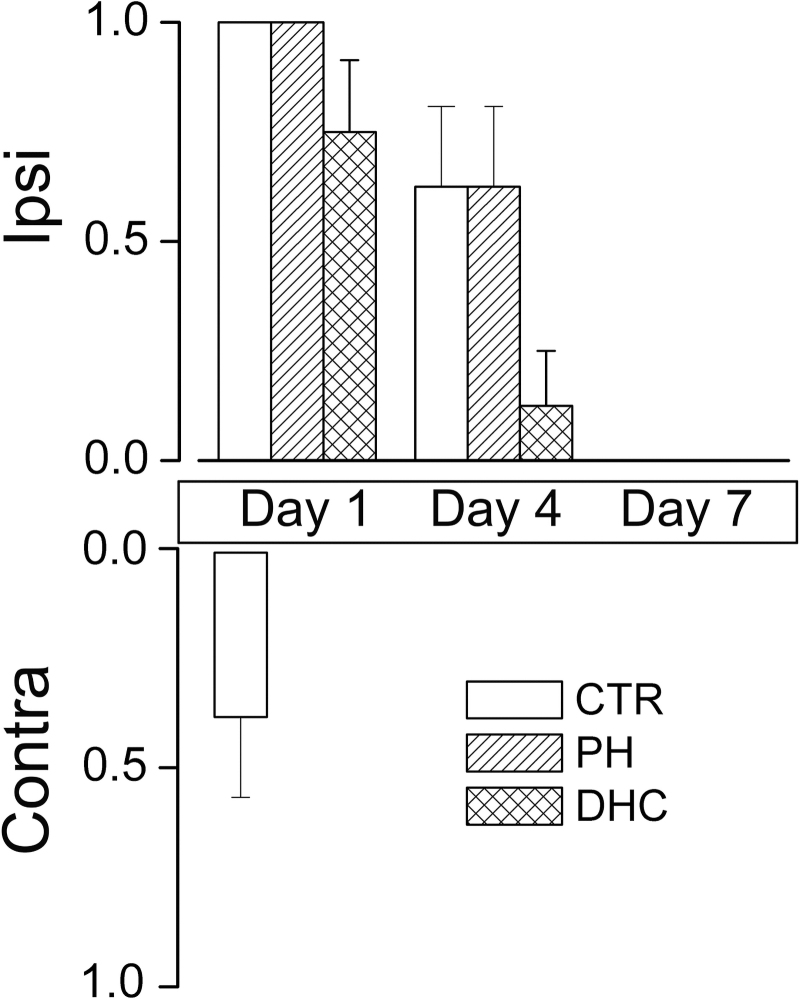
Ptosis after SCI. Incidence (mean ± SE) of ptosis involving the ipsilateral (ipsi) and contralateral (contra) eyes following lower cervical hemicord contusion in rats treated at 4 hours with normothermia (*empty bars*), with PH (*bars with diagonal hatches*), or with DHC-induced hypothermia (*bars with cross hatches*), on days 1, 4, and 7 following trauma; eight rats per group. DHC, dihydrocapsaicin; SCI, spinal cord injury; SE, standard error.

On day 1, ptosis was present ipsilaterally in most rats in all the three groups, whereas ptosis was present contralaterally only in some controls, but not in rats with hypothermia treatments (Fig. 3). On day 4, several rats still exhibited ipsilateral ptosis, but none had contralateral ptosis. By day 7, ptosis had resolved in all animals.

#### Urinary function

No animal developed urinary retention requiring Credé's maneuver to empty the bladder. However, several animals appeared to have incontinence early-on that resolved spontaneously. In all the three groups, on day 1, 2/8 animals had hematuria consistent with hemorrhagic cystitis, which may reflect involvement of the lateral funiculus (Ferrero *et al.*, 2014). In all cases, hematuria cleared by day 2 and was not observed at later times.

#### mBBB scores

**FIG. 4.**
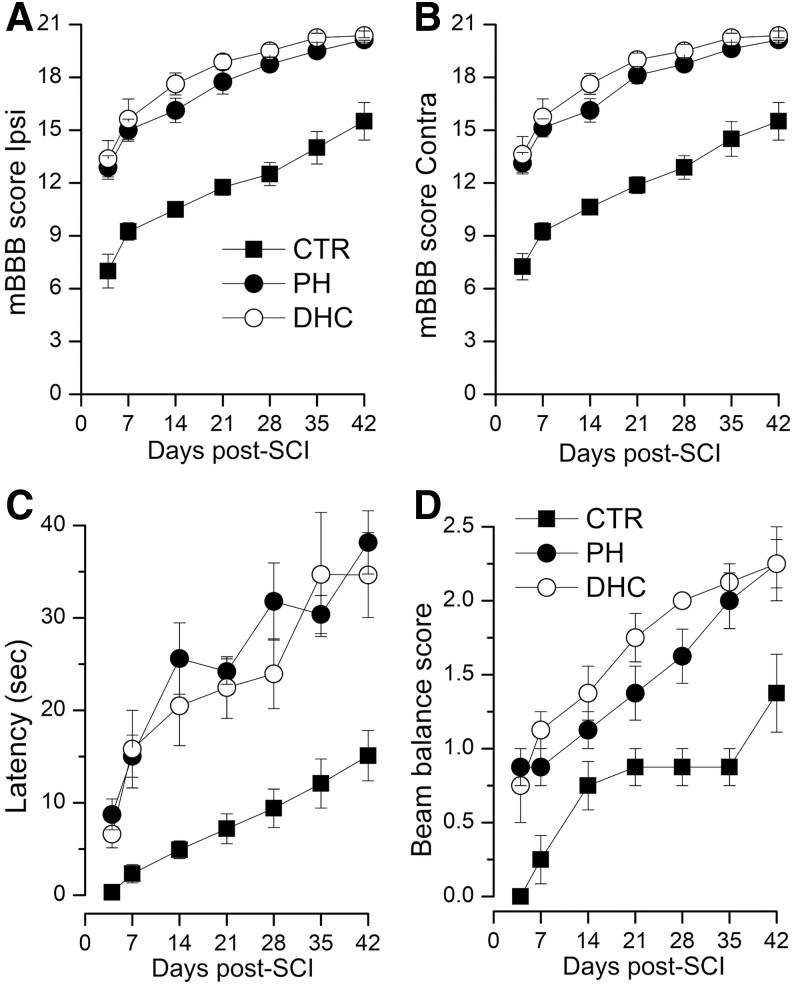
Motor function after SCI. **(A–D)** Ipsilateral **(A)** and contralateral **(B)** mBBB scores, performance on the accelerating rotarod **(C)**, and performance on the beam balance **(D)** in rats treated at 4 hours with normothermia (*filled squares*), with PH (*filled circles*), or with DHC-induced hypothermia (*empty circles*) during the 6 weeks after trauma; for all four panels, values for both hypothermia groups were significantly different from normothermic controls (repeated-measures ANOVA; *p* < 0.001), but ultimately were not different from each other; eight rats per group. ANOVA, analysis of variance; DHC, dihydrocapsaicin; mBBB, modified Basso, Beattie, and Bresnahan; PH, physically induced hypothermia; SCI, spinal cord injury.

Throughout the 6 weeks of testing, mBBB scores for both the hypothermia modalities were similar, showing progressive improvements with time of ipsilateral and contralateral hind limb function, with scores significantly better than normothermic controls (Fig. 4A, B). The maximum mBBB score of 21 in the ipsilateral hind paw was reached in rats with both the hypothermia modalities: in 1/8 versus 4/8 rats with physical versus DHC hypothermia (Fisher's exact test, two-sided; *p* = 0.28). No rat in the control group achieved an ipsilateral score of 21. BBB subscores at week 6, which are based on toe clearance, paw rotation, trunk stability, and tail up/down (Burke and Magnuson, 2012), were 6.6 ± 1.5, 12.1 ± 0.1, and 12.4 ± 0.4 for normothermic controls, physical hypothermia, and DHC-hypothermia, respectively. The subscores for both the hypothermia groups were statistically different from controls (*p* < 0.01) but were not different from each other.

#### Accelerating rotarod

This is a test of coordinated forelimb and hind limb function. Throughout the 6 weeks of testing, latencies for both the hypothermia modalities were similar, showing progressive improvements with time, with latencies significantly better than normothermic controls (Fig. 4C).

#### Beam balance

This also is a test of coordinated forelimb and hind limb function. Throughout the 6 weeks of testing, beam balance scores for both the hypothermia modalities showed progressive improvements with time, with scores significantly better than normothermic controls (Fig. 4D); during intermediate times, scores for the DHC-treated group were better, but by week 6, scores for the two modalities converged. No SCI rat achieved a score of 4 (walks on beam) typical of a neurologically intact normal rat.

#### Grip strength

**FIG. 5.**
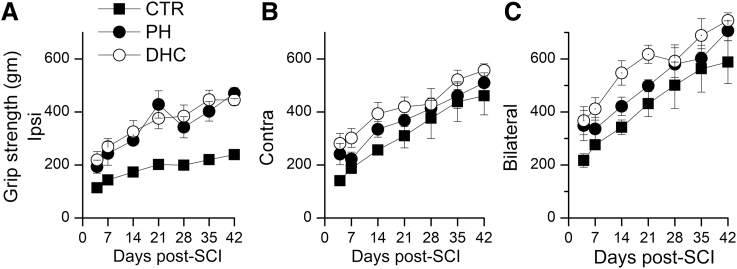
Hind limb grip strength after SCI. **(A–C)** Ipsilateral **(A)**, contralateral **(B)**, and combined ipsi- and contralateral **(C)** hind limb grip strength in rats treated at 4 hours with normothermia (*filled squares*), with PH (*filled circles*), or with DHC-induced hypothermia (*empty circles*) during the 6 weeks after trauma; for panel **(A)**, values for both hypothermia groups were significantly different from normothermic controls (repeated-measures ANOVA; *p* < 0.005); eight rats per group. ANOVA, analysis of variance; DHC, dihydrocapsaicin; PH, physically induced hypothermia; SCI, spinal cord injury.

The grip strength of the ipsilateral hind paw in rats treated with both the hypothermia modalities showed progressive improvements with time and was significantly better than that of normothermic controls (Fig. 5A). The distinction between treatments and controls was not as apparent on testing grip strength in the contralateral hind paw, or when the grip strength of both the hind paws together was tested (Fig. 5B, C).

#### Body mass

**FIG. 6.**
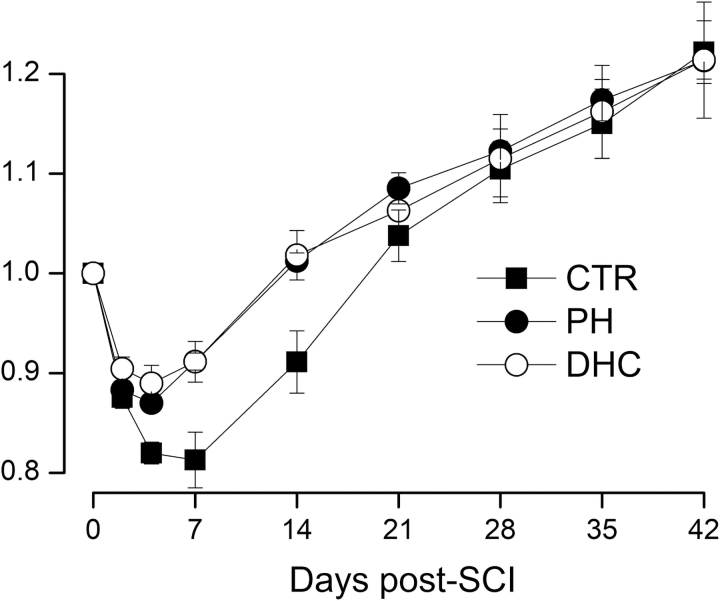
Body mass after SCI. Mass (mean ± SE), relative to preinjury baseline, following lower cervical hemicord contusion in rats treated at 4 hours with normothermia (*filled squares*), with PH (*filled circles*), or with DHC-induced hypothermia (*empty circles*) during the 6 weeks after trauma; values at days 4, 7, and 14 for both the hypothermia groups were significantly different from normothermic controls (repeated-measures ANOVA; *p* < 0.005); eight rats per group. ANOVA, analysis of variance; DHC, dihydrocapsaicin; PH, physically induced hypothermia; SCI, spinal cord injury; SE, standard error.

Normal weight gain in young adult rats is an important measure of general well-being, and is known to be impaired after SCI (Primeaux *et al.*, 2007; Hosier *et al.*, 2015). Body mass was measured on days 0, 2, 4, and 7 after trauma, and then weekly thereafter. Rats in all groups lost weight during the first week after trauma, but less with hypothermia treatments compared with controls (Fig. 6). During the first 3 weeks, body mass for both the hypothermia modalities differed from controls, but after week 3, body mass for the three groups converged.

#### Lesion size/spared tissue

**FIG. 7.**
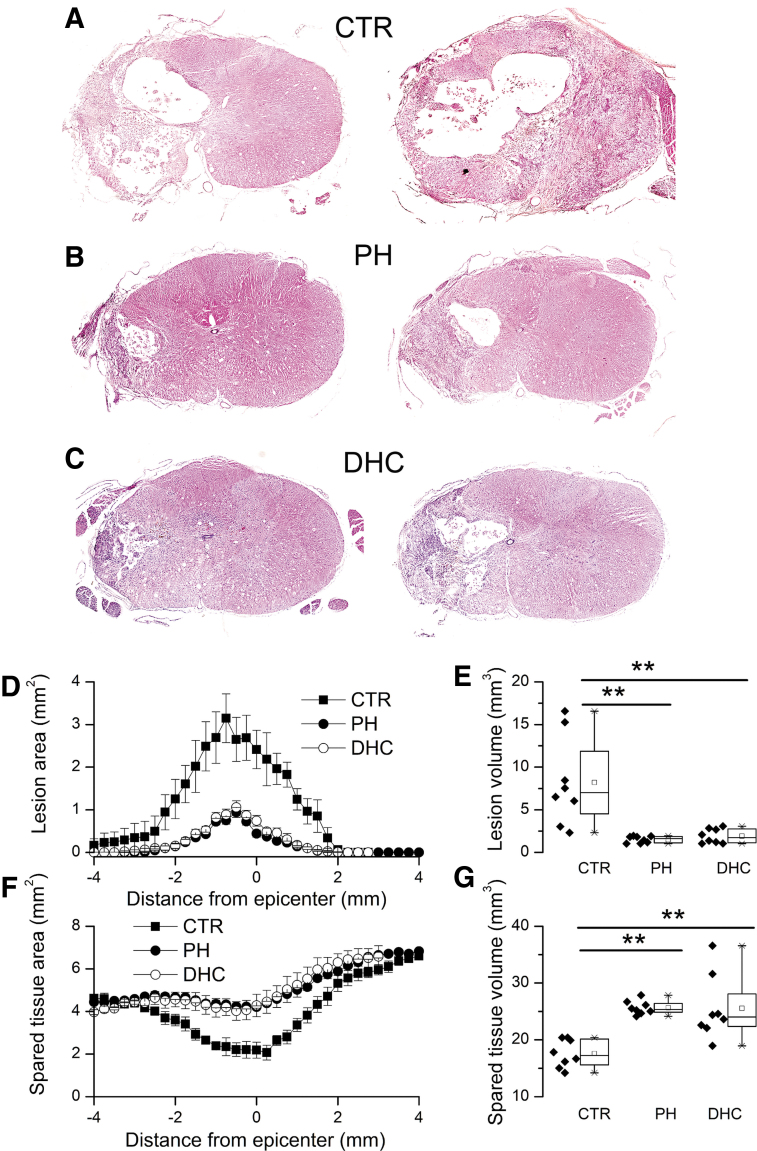
Lesion size and spared tissue after SCI. **(A–C)** H&E-stained sections of spinal cord at the epicenter of injury from two rats treated at 4 hours with normothermia **(A)**, with PH **(B)**, or with DHC-induced hypothermia **(C)**; note that the lesion induced unilaterally at the time of injury expanded to the contralateral side in the normothermic control but not in hypothermia-treated rats. **(D–G)** Area of lesion as a function of distance from epicenter **(D)**, lesion volume **(E)**, area of spared tissue as a function of distance from epicenter **(F)**, and volume of spared tissue **(G)** at 6 weeks following lower cervical hemicord contusion in rats treated at 4 hours with normothermia (*filled squares*), with PH (*filled circles*), or with DHC-induced hypothermia (*empty circles*); in **(D)** and **(F)**, areas for both the hypothermia groups were significantly different from normothermic controls (ANOVA; *p* < 0.001), but not from each other; in **(E)** and **(G)**, volumes for both hypothermia groups were significantly different from normothermic controls, but not from each other; eight rats per group; *p < 0.05; **p < 0.01. ANOVA, analysis of variance; DHC, dihydrocapsaicin; H&E, hematoxylin and eosin; PH, physically induced hypothermia; SCI, spinal cord injury.

After the 6-week period of testing, the spinal cords were analyzed to measure lesion volumes and spared spinal cord tissue. An important characteristic of the hemicord contusion model is that, when untreated, lesions typically expand across the midline (Fig. 7A) whereas, when successfully treated, contralateral expansion is reduced (Fig. 7B, C) (Simard *et al.*, 2012a). Here, the number of lesions that expanded to the contralateral side was 7/8, 1/8, and 1/8 for normothermic controls, rats with physical hypothermia, and rats with DHC hypothermia, respectively (*χ*^2^ = 12.8, *p* < 0.002).

Lesion areas were plotted as a function of distance from the epicenter (Fig. 7D), and were used to calculate volumes (Fig. 7E). Lesion volumes were as follows: 8.2 ± 1.8, 1.5 ± 0.1, and 1.9 ± 0.3 mm^3^ for normothermic controls, rats with physical hypothermia, and rats with DHC hypothermia, respectively.

Areas of spared spinal cord tissue were plotted as a function of distance from the epicenter (Fig. 7F), and were used to calculate volumes (Fig. 7G). Volumes of spared tissue were as follows: 17.6 ± 0.9, 25.7 ± 0.4, and 25.5 ± 2.0 mm^3^ for normothermic controls, rats with physical hypothermia, and rats with DHC hypothermia, respectively.

## Discussion

The principal finding of the present study is that pharmacologically induced hypothermia using DHC was as effective as physically induced hypothermia (PH) in SCI, as indicated by equally improved neurological function and lesion size/tissue preservation that in both cases were significantly better than normothermic controls. Moderate hypothermia induced by physical means has long been recognized to improve neurological function and to reduce lesion expansion, especially when initiated within 1 hour of the trauma (Batchelor *et al.*, 2013; Yousefifard *et al.*, 2020). Hypothermia instituted at the more clinically relevant times of 2 hours (Maybhate *et al.*, 2012; Teh *et al.*, 2018) or 4 hours (Wells and Hansebout, 1978; Hosier *et al.*, 2015) after trauma also has been shown to confer significant benefit. Previous work in a monkey SCI model suggested that 4 hours after trauma was the critical time window during which cooling needed to be initiated (Albin *et al.*, 1968; White *et al.*, 1969). Here, we studied outcomes when moderate systemic hypothermia, both physical and pharmacological, was initiated at 4 hours after traumatic SCI. Both modes of hypothermia induction showed significant benefit when initiated at 4 hours, with no discernable difference between modes of induction in terms of neurological function or lesion size/tissue preservation. We did not study the effect of the two modes of induction administered together, although in models of cerebral ischemia, there is evidence that the two together may act synergistically (Wu *et al.*, 2017; Zhang *et al.*, 2018).

TRPV1 agonists such as DHC are among the most widely studied drugs for inducing hypothermia pharmacologically. Fosgerau *et al.* (2010b) screened a heterogeneous group of TRPV1 agonists and tested their hypothermic properties, finding that DHC best displayed the desired hypothermic profile with regard to duration, depth, and control. Continuous infusion of DHC induced sustainable and clinically relevant mild hypothermia in rats, cynomolgus monkeys, and young cattle (71–86 kg calves). The experiments with calves showed that IV infusion of DHC could maintain mild hypothermia (−3°C or more) for more than 12 hours, a result that is important because, unlike laboratory rodents, large animals and humans have a surface area-to-mass ratio that is relatively small, which favors heat retention. DHC has been used to induce pharmacological hypothermia in animal models of cardiac arrest (Junyun *et al.*, 2016; Zhong *et al.*, 2018) and cerebral ischemia (Cao *et al.*, 2014, 2017; Janyou *et al.*, 2017) with favorable results, but had not been examined previously in models of SCI.

The mechanism of neuroprotection with DHC is presumably linked to hypothermia, but other effects of DHC also must be acknowledged. In normal animals, DHC infusion causes initial tachycardia and hypertension and, at high doses (>2.0 mg/kg/hour), causes episodes of transient bradycardia and hypotension, consistent with a Bezold/Jarisch reflex (Fosgerau *et al.*, 2010a). Also in normal animals, DHC depletes substance P from peptidergic nerve fibers, dorsal root ganglia, and dorsal spinal cord, and it alters mast cell density in the respiratory tract (Miller *et al.*, 1982; Ramirez-Romero *et al.*, 2000). When studied *in vitro*, under conditions in which lowered temperature cannot contribute, DHC acts as an antioxidant (Rosa *et al.*, 2002; Nascimento *et al.*, 2014) and it inhibits *in vitro* platelet aggregation and thromboxane B2 formation (Adams *et al.*, 2009; Almaghrabi *et al.*, 2014, 2018). In models of focal (Cao *et al.*, 2014, 2017; Janyou *et al.*, 2017, 2020; Wu *et al.*, 2017) and global (Junyun *et al.*, 2016; Zhong *et al.*, 2018) cerebral ischemia, several biochemical effects of DHC have been reported that are difficult to disentangle from hypothermia *per se.* Critically, however, the salutary effects of DHC in ischemia are not observed in TRPV1 knockout mice, strongly implicating TRPV1 as a principal mechanism of action (Cao *et al.*, 2014). For DHC in SCI, the relative contribution, if any, of alternative, noncooling mechanisms remains to be determined.

Excellent reviews have been published examining the various mechanisms by which hypothermia may act (Ahmad *et al.*, 2014; Wang and Pearse, 2015; Alkabie and Boileau, 2016). The neuroprotective mechanisms of hypothermia are yet to be fully elucidated, and may differ in central nervous system (CNS) ischemia versus trauma. In models of ischemia, hypothermia reduces energy metabolism (oxygen consumption and glycolysis) (Zager and Ames, 1988; Allen *et al.*, 1994; Quinones-Hinojosa *et al.*, 2003), excitotoxicity (Ishikawa and Marsala, 1999; Wakamatsu *et al.*, 1999), and other pathological mechanisms. In studies specifically on models of traumatic SCI, hypothermia reduces spinal cord blood flow (Westergren *et al.*, 2001), vasogenic edema and spinal cord swelling (Yu *et al.*, 1999), inflammation (IL-1β, NOS2, neutrophil invasion, and microglial/macrophage activation) (Chatzipanteli *et al.*, 2000; Dietrich *et al.*, 2004; Ha and Kim, 2008; Morino *et al.*, 2008; Kao *et al.*, 2011), oxidative stress and lipid peroxidation (Tuzgen *et al.*, 1998; Luo *et al.*, 2002; Duz *et al.*, 2009; Topuz *et al.*, 2010; Karamouzian *et al.*, 2015), and neuronal death, apoptosis, and autophagy (Shibuya *et al.*, 2004; Ha and Kim, 2008; Lo *et al.*, 2009; Kao *et al.*, 2011; Ok *et al.*, 2012; Grulova *et al.*, 2013; Seo *et al.*, 2015). In SCI, hypothermia also may be associated with reduced excitotoxicity (glutamate), although the literature on this is limited and mixed (Farooque *et al.*, 1997; Yamamoto *et al.*, 1998). In addition, in SCI, hypothermia promotes angiogenesis and neurogenesis (Kao *et al.*, 2011).

Arguably, one of the most important mechanisms by which hypothermia improves outcome after traumatic SCI is by reducing lesion expansion. Traumatic impact to the spinal cord causes shearing of blood vessels that leads to a primary hemorrhage proportional to the severity of impact (Balentine, 1978). This is followed within hours by a two- to threefold volumetric expansion of the lesion, as an evolving microvascular dysfunction causes a progressive conversion of normal to abnormal tissue, an autodestructive process termed “progressive hemorrhagic necrosis” (PHN) (Simard *et al.*, 2007, 2010; Gerzanich *et al.*, 2009; Fassbender *et al.*, 2011). Acute lesion expansion has been documented using serial magnetic resonance imaging in both rodents with experimental SCI (Bilgen *et al.*, 2000; Simard *et al.*, 2013) and humans with SCI (Aarabi *et al.*, 2012, 2017). In animal models of SCI, lesion volume correlates with neurological function (Patel *et al.*, 2016), and treatments that reduce lesion volume are associated with better neurological outcomes (Gerzanich *et al.*, 2009; Simard *et al.*, 2010; Hosier *et al.*, 2015). In humans with SCI, lesion volume strongly impacts outcome, including the degree of recovery (Aarabi *et al.*, 2012, 2017).

The effect of hypothermia on lesion volume is likely a critical determinant of outcome after SCI. In various studies, hypothermia has been reported to reduce lesion volumes by 32–47% (Yu *et al.*, 2000; Lo *et al.*, 2009; Batchelor *et al.*, 2010). In the model studied here, we previously reported a 35% reduction (4.8–3.1 mm^3^) in volume (Hosier *et al.*, 2015). In the present study, both pharmacological and physical hypothermia reduced lesion volume several folds, at both 48 hours and 6 weeks. Many of the salutary effects reviewed above that are characteristic of hypothermia, including reductions in spinal cord swelling, inflammation, oxidative stress, neuronal death, and others, could simply be due to a reduction in lesion volume.

The molecular mechanism that underlies PHN and lesion expansion involves transcriptional upregulation of SUR1-TRPM4 in microvessels. Inhibition of SUR1-TRPM4 by either genetic or pharmacological means reduces PHN and lesion expansion (Gerzanich *et al.*, 2009; Simard *et al.*, 2010). We previously reported that, in a direct comparison, hypothermia and pharmacological inhibition of SUR1-TRPM4 were equally effective at reducing lesion volume and improving neurological outcome (Hosier *et al.*, 2015). Although not evaluated in SCI, in other pathological conditions associated with increased levels of SUR1-TRPM4, hypothermia reduces both mRNA and protein levels of *Abcc8*/SUR1 and *Trpm4*/Trpm4 (Huang *et al.*, 2016; Nakayama *et al.*, 2018). Thus, it is plausible that the reduction in lesion expansion with hypothermia could be linked to hypothermia-induced inhibition of SUR1-TRPM4 expression.

This study has limitations. We did not examine the effect of administering vehicle alone (2% DMSO), and DMSO can be protective in SCI. The total dose of DMSO that we administered was 59 mg/kg, one time over 4 hours, beginning 4 hours after trauma. However, a dose of 110 mg/kg was shown previously to have no significant salutary effect in experimental SCI (Turan *et al.*, 2008), and a dose of 1–2 gm/kg is reportedly required for protection in SCI (Kajihara *et al.*, 1973; de la Torre *et al.*, 1975; Gelderd *et al.*, 1980, 1983; Goodnough *et al.*, 1980) (see review [Jacob and de la Torre, 2009]). Based on this, it seems unlikely that the benefit that we observed with DHC in 2% DMSO could be attributed to DMSO. Also, our initial series of experiments on long-term outcomes were designed specifically to allow direct comparison of the two modes of hypothermia, not hypothermia versus normothermia, and as a result, the normothermic controls for this experiment were not carried out contemporaneously. This experimental design raises the possibility of bias in terms of comparing hypothermia versus normothermia. However, the results obtained in the present study in terms of mBBB and lesion size were in good agreement with the results published previously with hypothermia treatment in this model (Hosier *et al.*, 2015). Moreover, our experiment here with short-term outcomes on mBBB and lesion size, conducted with the three groups studied randomly and contemporaneously, yielded results that were comparable with the experiment with long-term outcomes. Finally, although it is known that high or repeated doses of capsaicin can induce an initial pain sensation that is followed by analgesia (see review (Fattori *et al.*, 2016)), we did not evaluate IV DHC for possible undesirable irritant effects or for potential worsening of postsurgical pain, which could limit its utility.

In summary, when administered at 4 hours after traumatic SCI, DHC-induced hypothermia and PH were equally effective at improving neurological function and reducing lesion volume, compared with normothermic controls. Although comparable in efficacy, DHC-induced hypothermia was less cumbersome to implement, and can be used outside of a hospital setting. One advantage of DHC is that, when administered IV, it is metabolized very rapidly (Rollyson *et al.*, 2014), facilitating precise adjustments of body temperature. Another advantage is that cooling-induced shivering and tachycardia, which are prominent with PH, are suppressed by DHC (Feketa *et al.*, 2013). At present, studies on IV DHC in humans are limited (Arndt *et al.*, 1993), with no Phase 1 study available to gauge clinical feasibility. Nevertheless, the possibility of inducing hypothermia in the field, at the site of trauma, is highly attractive, since it would facilitate early and rapid induction of hypothermia for optimizing outcome after traumatic SCI.
